# Editorial: Shared Autonomy— Learning of Joint Action and Human-Robot Collaboration

**DOI:** 10.3389/fnbot.2019.00016

**Published:** 2019-05-14

**Authors:** Malte Schilling, Wolfram Burgard, Katharina Muelling, Britta Wrede, Helge Ritter

**Affiliations:** ^1^Center of Excellence ‘Cognitive Interaction Technology’, Bielefeld University, Bielefeld, Germany; ^2^Department of Computer Science at the University of Freiburg, Freiburg, Germany; ^3^Robotics Institute, Carnegie Mellon University, Pittsburgh, PA, United States

**Keywords:** shared autonomy, human-robot interaction, learning, collaboration, interactive robots

Advancing the autonomy of artificial agents, such as robots, comes with an important challenge: how to shape the autonomy of an individual agent in such a manner that bringing several such agents together leads to a suitable fusion or sharing of (parts of) their individual autonomy spaces. This challenge has opened up important new perspectives on how to make robots more skillful, versatile, and easier to deploy for tasks and scenarios where robots can no longer act solitarily.

The special issue aims at, first, providing a theoretical perspective to describe autonomy in collaborative settings, and, secondly, presenting novel results that highlight common traits and underlying dimensions and can guide further developments on adaptive interaction in teams of humans and robots.

## Dimensions of Shared Autonomy

**Figure 1:**
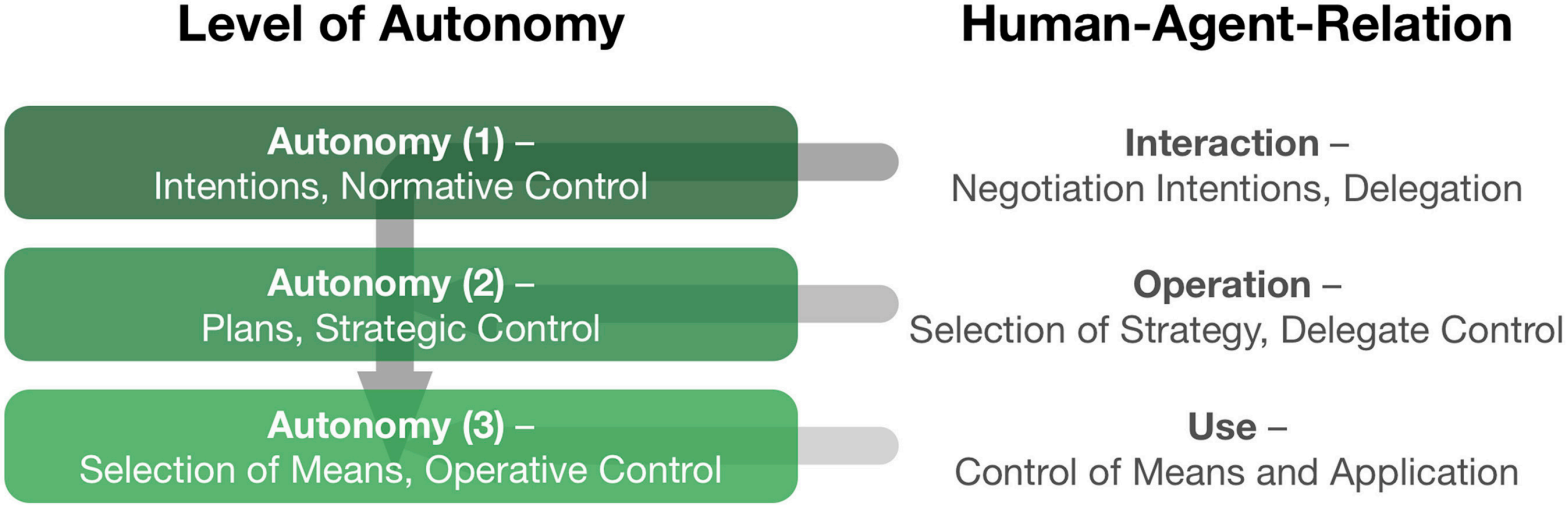
Distinguishing multiple levels of autonomy (shown on the left). Conceptually, these different levels characterize the freedom of decision making arising at different levels of an abstraction hierarchy. The notion of Shared Autonomy allows to analyze and design types of interaction patterns between human users and other agents (shown on the right). For details see Schilling et al. (2016).

Accordingly, Shared Autonomy has to address the question on how to mediate autonomy between participating actors. This question can be approached through a distinction of different levels of autonomy and by defining characteristic dimensions describing collaborative scenarios (see Figure 1 and Schilling et al., 2016). In their review, Alonso and de la Puente identify transparency as one such characteristic which is understood as observability and predictability. They show that this translates well to interactive multi-agent scenarios and provides useful dimensions to account for interaction patterns. As predictability decreased in more complex tasks, systems were found to act more autonomously. They reasoned that this is due to limited observability which can be counteracted by subsuming low-level details in higher-level representations as are goals. Here, Gildert et al. continue with their review on joint action which highlights a shared context as a requirement for prediction. Furthermore, they point out the role of attention and how implicit and explicit communication can guide attention between agents in order to mediate interaction patterns. Both articles connect the issue of shared information in a further step toward the notion of trust in interactive settings.

## Shared Autonomy for Human-Robot Collaboration

The special issue also provides examples that highlight current advances in human-robot collaboration and show that different levels of a representational hierarchy may call for different mechanisms and strategies: lower levels deal with the optimal selection of means to achieve immediate goals; autonomy on an intermediate level determines strategies that fulfill higher level purposes which are given on the highest level (as intentions). Importantly, studies on coordination with robots on a higher-level further stress that this can't be addressed solely on an abstract level, but always requires grounding in lower action levels. This further emphasizes the importance of a differentiated perspective on Shared Autonomy.

For coordination on the lower level of immediate action, Trinh et al. provide a solution to navigation for multiple agents as a prototypical example for Shared Autonomy scenarios. Each agent preplans its own route to a target location which is represented as a static flow field. To avoid collisions, this representation is overlaid with a constantly updated dipole flow field of other agents' locations. The agents' behavior is mostly driven by the preplanned flow field, but the dipole flow field shapes the routes of the agents autonomously. Ewerton et al. directly address this change of control and autonomy. Their framework deals with visual and haptic feedback to users while learning motor skills. In their case users are intended to learn drawing Japanese characters. Probabilistic models of skills are learned following a reinforcement learning approach and the skill likelihood is used to regulate the amount of feedback. The system autonomously steps in when needed and starts to provide guidance through haptic feedback.

The focus of Akkaladevi et al. is on how cooperation can be organized between multiple agents in a collaborative assembly process. On the lowest level, this entails learning basic actions. On a higher level, the system aims at putting these into a sequence. In addition, the system attempts to propose suitable actions that are chosen depending on background knowledge about users' capabilities, current context and current goals. Knowledge was learned using incremental reinforcement learning. The Interactive Shared Solution Shaping paradigm by Reardon et al. focuses further on an intermediate level of representation for negotiating a shared plan: while the autonomous agent has its own planning process, a human user provides feedback based on his expert domain knowledge. This was realized for planning the route of a surveillance robot. While the user doesn't need to know all the details about, e.g., collision avoidance, mediation of the planning process requires transparency between the robot system and the user. A form of interaction is needed that details current routes to the user and allows to influence the (re)planning process. Establishing such a form of fast and reliable communication is crucial for systems that should work autonomously, but at the same time contribute toward a user's intention and provide valuable information. In Shukla et al. such multimodal coordination is further analyzed and applied. They introduce the probabilistic Proactive Incremental Learning framework that learns to associate hand gestures with manipulation actions in a collaborative assembly task. There, communication is required to establish common ground and align mutual beliefs between robot and user which exploits information about gaze. The system anticipated users' behaviors and goals in order to proactively assist the user. In a study with non-roboticist users they found that their proactive system reduced the interaction effort and was mostly preferred, but further cautioned that this requires trust.

In a further user study, Schulz et al. turn toward the question of how preferred interaction with robots is task dependent. They categorize forms of collaboration along two dimensions: first, distinguishing independent actions and joint interaction; secondly, distinguishing sequences where order is crucial versus not. This was assessed in a series of table-top scenarios in which a human collaborated with a robot to build a given design in a blocks world. They found that mostly autonomous actions of the robot are more efficient and preferred.

## Conclusion

The special issue brings together current work and trends in Shared Autonomy. Collaboration in teams of humans and robots shifts more and more toward higher level tasks that not only include low level coordination of immediate action. This requires coordination of complex plans between multiple agents that can be adapted at runtime. Therefore, each agent is tasked with autonomous control on different levels, but also has to respect the autonomy of other agents and therefore has to adjust its degree of autonomy. Shared Autonomy provides a useful perspective on the underlying interaction patterns, how these are adapted and what requirements this poses to systems, like establishing common ground, transparency and shared beliefs as well as goals, trust, and efficient forms of communication.

## Author Contributions

MS prepared the figure and wrote the paper. HR, WB, KM, and BW discussed and wrote the paper.

### Conflict of Interest Statement

The authors declare that the research was conducted in the absence of any commercial or financial relationships that could be construed as a potential conflict of interest.
